# Prognostic significance of dihydropyrimidine dehydrogenase expression in breast cancer

**DOI:** 10.1038/sj.bjc.6600040

**Published:** 2002-01-21

**Authors:** J Horiguchi, H Takei, Y Koibuchi, K Iijima, J Ninomiya, K Uchida, R Ochiai, M Yoshida, T Yokoe, Y Iino, Y Morishita

**Affiliations:** Second Department of Surgery, Gunma University Faculty of Medicine, Showa-machi 3-39-15, Maebashi, Gunma 371-8511, Japan; Department of Emergency and Critical Care Medicine, Gunma University Faculty of Medicine, Showa-machi 3-39-15, Maebashi, Gunma 371-8511, Japan

**Keywords:** breast cancer, 5-FU, DPD, immunohistochemistry, prognosis

## Abstract

We have investigated dihydropyrimidine dehydrogenase expression as a prognostic marker in breast cancer. A total of 119 women with breast cancer undergoing surgery between 1985 and 1996 were included in this study. Eighty-seven patients were treated with postoperative chemotherapy including 5-fluorouracil or 5-fluorouracil derivatives. Fifty-nine (50%) of 119 patients were determined to be immunostaining-positive for dihydropyrimidine dehydrogenase. There was no significant difference between dihydropyrimidine dehydrogenase staining and tumour size, lymph node status, clinical stage, oestrogen receptor status, histologic grade, or 5-fluorouracil administration. When evaluated in patients treated with 5-fluorouracil or 5-fluorouracil derivatives, patients with dihydropyrimidine dehydrogenase-positive tumours had a significantly (*P*<0.05) poorer disease-free survival compared to those with dihydropyrimidine dehydrogenase-negative tumour. No conclusion can be drawn about the prognostic impact of dihydropyrimidine dehydrogenase status in patients who were not treated with 5-fluorouracil regimes due to the small number of such cases in this series. Lymph node and dihydropyrimidine dehydrogenase status were independent prognostic factors for disease-free survival, and lymph node status for overall survival using multivariate analysis. In conclusion, dihydropyrimidine dehydrogenase is a possible prognostic factor in patients with breast cancer treated with 5-fluorouracil or 5-fluorouracil derivatives.

*British Journal of Cancer* (2002) **86**, 222–225. DOI: 10.1038/sj/bjc/6600040 www.bjcancer.com

© 2002 The Cancer Research Campaign

## 

5-fluorouracil (5-FU) has been widely used in the treatment of breast cancer, either singly or in combination with other cytostatics. 5-FU is initially catabolized to 5-fluorodihydrouracil by dihydropyrimidine dehydrogenase (DPD), mainly in the liver, and then dihydropyrimidinase and β-ureido-propionase catalyze the formation of 2-fluoro-β-alanine. More than 80% of an administered dose of 5-FU is eliminated by catabolism through DPD (Milano and McLeod, 2000). A high level of tumoral DPD metabolizes 5-FU to inactive products before cytotoxic nucleotides can be formed. Although resistance to 5-FU is multifactorial, tumoral DPD activity may influence the efficacy of 5-FU. Several studies have demonstrated that high DPD levels result in low sensitivity to 5-FU (Ishikawa *et al*, 2000; Salonga *et al*, 2000; Inada *et al*, 2000; Mizutani *et al*, 2001).

Immunohistochemistry has the advantage of permitting the evaluation of protein expression *in situ* using paraffin-embedded blocks of specimens. There are only a few publications which discuss immunohistochemical evaluation of DPD levels (Huang *et al*, 2000; Miyamoto *et al*, 2000; Takenoue *et al*, 2000). Takenoue *et al* (2000) investigated DPD expression using immunohistochemistry in colon carcinoma, and demonstrated that immunohistochemical score was correlated with protein levels of DPD.

In this study, we performed an immunohistochemical study of intratumoral DPD expression in breast cancer, and examined the prognostic and predictive significance of DPD expression in breast cancer patients.

## PATIENTS AND METHODS

A total of 119 women with breast cancer undergoing surgery between 1985 and 1996 were studied. The age at the time of surgery ranged from 30 to 85 years (median, 51). Pathological examination revealed that axillary lymph node status was positive in 56 patients and negative in 63. The tumour size of the resected specimen was 2 cm or less in 23 patients and larger than 2 cm in 96 patients. Oestrogen receptor (ER) content of tumours was measured using an enzyme immunosorbent assay. Of 119 patients, 44 were ER-positive, 62 were ER-negative, and 13 were ER-unknown. Histologic grading was performed by combining cell morphology, tissue architecture, and assessment of the cell proliferation rate (Bloom and Richardson, 1957). Histologic grade was known for 116 patients with invasive breast carcinomas. Thirty-two patients (28%) grade I, 57 (49%) had grade II and 27 (23%) had grade III tumours. 5-FU-based chemotherapy was administered to patients with vascular invasion-positive tumours in the 1980s and all patients in the 1990s. Eighty-seven patients were treated with postoperative chemotherapy including 5-FU or 5-FU derivatives. Sixty-four patients received 20 mg of tamoxifen for at least 2 years. Immunohistochemical examination was conducted using 4 μm of sections taken from formalin-fixed, paraffin-embedded blocks. Immunostaining was performed on the sections using an Envision system (Dako Japan Co., Kyoto). After deparaffinization with xylene and hydration with downgraded ethanol, the sections were incubated in 0.3% H_2_O_2_ in methanol for 5 min at room temperature. The rhDPD polyclonal antibody (generously supplied by Dr Fukushima, Taiho Pharmaceutical Co., Ltd.) was used as a primary antibody.

**Figure 1:**
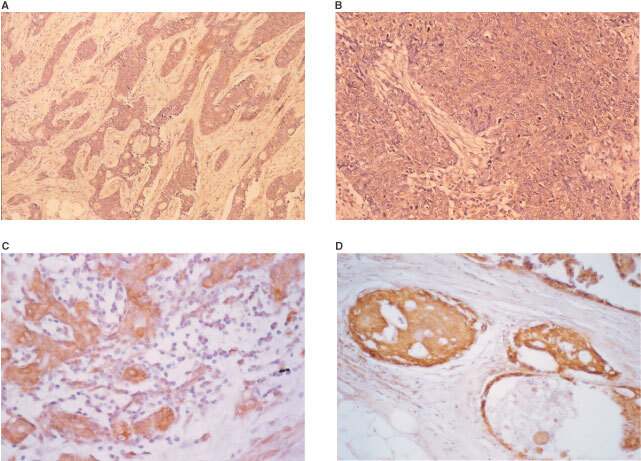
Immunohistochemical staining for DPD. (**A**) Grade 0 (no staining), (**B**) grade 2 (weak), (**C**) grade 3 (intermediate), (**D**) grade 3 (strong).

This is an antibody highly specific against rhDPD expressed in the baculovirus-expression system using human DPD cDNA. The expressed rhDPD protein has been found to retain the entire molecular form and to show a high 5-FU-degrading activity equivalent to that of the human liver DPD. Using this recombinant protein, polyclonal antibody was generated and investigated for its specificity, relationship to enzyme activity and the possibility of immunohistochemical measurement of tumoral DPD. The polyclonal antibody reacted with both human and rodent DPD. Tumour cells expressing high levels of DPD showed strongly positive staining, but those expressing low level or no DPD showed no staining (Okabe *et al*, 2000). The sections were rinsed with a buffer solution, and peroxidase labelled polymer was incubated for 30 min at room temperature. After the sections were washed with a buffer solution, the peroxidase reaction developed with diaminobenzidine tetrahydrochloride, and nuclei were counterstained with haematoxylin. DPD staining was noted in the cytoplasm of the cancer cells. DPD staining results were classified into four grades (0: no staining, 1: weak, 2: intermediate, and 3: strong) according to the intensity (Figure 1A, B, C and D

). Immunostaining of grades 0 and 1 was determined as DPD negative and grades 2 and 3 as positive. MIAPaCa-2 cells were used as positive control.

## RESULTS

Of the 119 patients evaluated, 59 (50%) of 119 patients showed immunostaining-positive for DPD. There was no significant difference between DPD staining and tumour size, lymph node status, clinical stage, ER status, histologic grade, or 5-FU and/or tamoxifen administration (Table 1

**Table 1:**
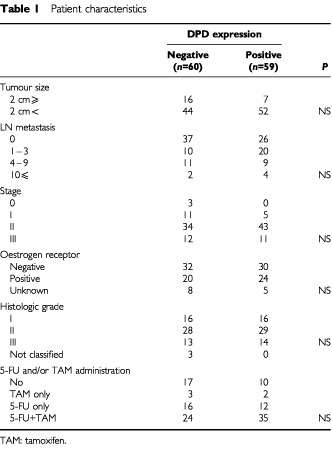
Patient characteristics

). The follow-up period ranged from 5 to 126 months (median 66 months). Actuarial disease-free survival and overall survival were calculated using the Kaplan-Meier method. Patients with DPD-positive tumours had a significantly (*P*<0.05) poorer prognosis in disease-free survival and overall survival compared to those with DPD-negative tumours (Figures 2

**Figure 2:**
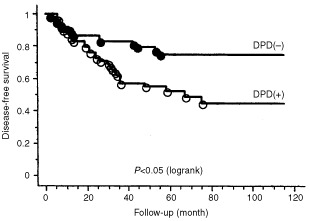
Disease-free survival by DPD expression. Patients with DPD-positive tumour had a significantly (*P*<0.05) poorer disease-free survival than those with DPD-negative tumour.

and 3

**Figure 3:**
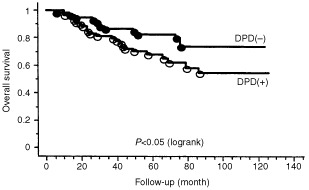
Overall survival by DPD expression. Patients with DPD-positive tumour had a significantly (*P*<0.05) poorer overall survival than those with DPD-negative tumour.

). When evaluated in patients treated with 5-FU or 5-FU derivatives, DPD expression was a significantly (*P*<0.05) poorer prognostic factor in disease-free survival (Figure 4

**Figure 4:**
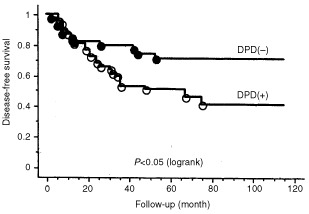
Disease-free survival by DPD expression in patients treated with 5-FU or 5-FU derivatives. Patients with DPD-positive tumour had significantly (*P*<0.05) poorer disease-free survival than those with DPD-negative tumour in patients treated with 5-FU or 5-FU derivatives.

). The small number of cases in this series who were not given 5-FU based chemotherapy means that no conclusion can be drawn about the prognostic effect of DPD status in this group of better prognostic patients. Multivariate analysis demonstrated that DPD and lymph node status were independent prognostic factors for disease-free survival. Lymph node status was an independent prognostic factor for overall survival. (Tables 2

**Table 2:**
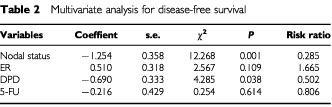
Multivariate analysis for disease-free survival

and 3

**Table 3:**
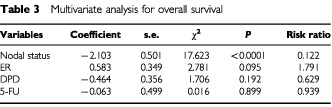
Multivariate analysis for overall survival

).

## DISCUSSION

DPD demonstrates variable activity in human tumours and the variation in tumoral DPD may influence the efficacy of 5-FU. A significant linear correlation has been observed between DPD activity and 5-FU clearance in patients with head and neck cancer while simultaneously monitoring 5-FU pharmacokinetics during a 5-day, continuous infusion 5-FU therapy (Fleming *et al*, 1992). Tumoral DPD is associated with tumour response to 5-FU in patients with gastric cancer (Inada *et al*, 2000; Ishikawa *et al*, 2000), colorectal cancer (Salonga *et al*, 2000), or bladder cancer (Mizutani *et al*, 2001). Little is known about the relationship between DPD level and cancer characteristics. Mizutani *et al* (2001) evaluated DPD activity in bladder cancer. The levels of DPD activity in grade 2 and grade 3 bladder cancers were approximately three-fold and four-fold higher than that in grade 1 cancers, respectively (Mizutani *et al*, 2001). Our study however did not show any relationship between DPD level and clinicopathological factors including histologic grade breast cancer. Takabayashi *et al* (2000) reported that neither tumour depth nor lymph node metastases was correlated with DPD activity in patients with gastric cancer. Huang *et al* (2000) also reported no correlation between intratumoral DPD staining and tumour status, nodal status, smoking habits, tumour differentiation and tumour histology in non-small cell lung cancer.

The prognostic significance of DPD expression in breast cancer has not been completely investigated. Huang *et al* (2000) evaluated intratumoral expression of DPD in non-small cell lung cancer immunohistochemically, and the 5-year survival rate of patients with high-DPD tumours was significantly lower than for patients with low-DPD tumours. Our immunohistochemical study clarified the prognostic significance of the DPD expression in breast cancer. Although DPD has the highest activity in liver and mononuclear cells, DPD expression in tumours has demonstrated itself to be a useful marker for disease-free survival in breast cancer patients treated with 5-FU-based chemotherapy. Even if DPD activity in the liver is low, a high level of tumour DPD would metabolise 5-FU to inactive products before cytotoxic nucleotides could be formed. On the other hand, DPD expression showed no prognostic function in patients who received no 5-FU-based chemotherapy. The number of patients evaluated was too small to preclude the prognostic function of DPD in patients who received no 5-FU-based chemotherapy. Moreover, the patients who received no 5-FU-based chemotherapy showed good prognosis irrespective of DPD levels, since the patients with vascular invasion-negative tumours were not treated with 5-FU-based chemotherapy in follow-up.

DPD inhibition has become a major goal in the strategy for the development of 5-FU treatment. A new antitumour agent based on biochemical modulation of 5-FU (S-1), consisting of tegafur (FT), 5-chloro-2, 4-dihydroxypyridine (CDHP), and potassium oxonate (Oxo) in a molar ratio of 1:0.4:1 has recently been developed (Shirasaka *et al*, 1996; Hirata *et al*, 1999). FT is a prodrug of 5-FU, and CDHP competitively inhibits DPD about 180 times more effectively than uracil, and leads to the retention of a prolonged concentration of 5-FU. 5-FU or its derivatives used in this study did not improve the prognosis of patients with DPD-positive tumours. Our results suggest that a strong DPD inhibitor with 5-FU based chemotherapy, for example S-1 treatment, should be considered to improve the prognosis of breast cancer patients with DPD-positive tumours.
